# *INK4/ARF* Transcript Expression Is Associated with Chromosome 9p21 Variants Linked to Atherosclerosis

**DOI:** 10.1371/journal.pone.0005027

**Published:** 2009-04-03

**Authors:** Yan Liu, Hanna K. Sanoff, Hyunsoon Cho, Christin E. Burd, Chad Torrice, Karen L. Mohlke, Joseph G. Ibrahim, Nancy E. Thomas, Norman E. Sharpless

**Affiliations:** 1 Department of Genetics, The Lineberger Comprehensive Cancer Center, The University of North Carolina School of Medicine, Chapel Hill, North Carolina, United States of America; 2 Department of Medicine, The Lineberger Comprehensive Cancer Center, The University of North Carolina School of Medicine, Chapel Hill, North Carolina, United States of America; 3 Department of Biostastisitcs, The Lineberger Comprehensive Cancer Center, The University of North Carolina School of Medicine, Chapel Hill, North Carolina, United States of America; 4 Department of Dermatology, The Lineberger Comprehensive Cancer Center, The University of North Carolina School of Medicine, Chapel Hill, North Carolina, United States of America; Ordway Research Institute, United States of America

## Abstract

**Background:**

Genome-wide association studies (GWAS) have linked common single nucleotide polymorphisms (SNPs) on chromosome 9p21 near the *INK4/ARF* (*CDKN2A/B*) tumor suppressor locus with risk of atherosclerotic diseases and type 2 diabetes mellitus. To explore the mechanism of this association, we investigated whether expression of proximate transcripts (*p16^INK4a^*, *p15^INK4b^*, *ARF*, *ANRIL* and *MTAP*) correlate with genotype of representative 9p21 SNPs.

**Methodology/Principal Findings:**

We analyzed expression of 9p21 transcripts in purified peripheral blood T-cells (PBTL) from 170 healthy donors. Samples were genotyped for six selected disease-related SNPs spanning the *INK4/ARF* locus. Correlations among these variables were determined by univariate and multivariate analysis. Significantly reduced expression of all *INK4/ARF* transcripts (*p15^INK4b^*, *p16^INK4a^*, *ARF* and *ANRIL*) was found in PBTL of individuals harboring a common SNP (rs10757278) associated with increased risk of coronary artery disease, stroke and aortic aneurysm. Expression of *MTAP* was not influenced by rs10757278 genotype. No association of any these transcripts was noted with five other tested 9p21 SNPs.

**Conclusions/Significance:**

Genotypes of rs10757278 linked to increased risk of atherosclerotic diseases are also associated with decreased expression in PBTL of the *INK4/ARF* locus, which encodes three related anti-proliferative transcripts of known importance in tumor suppression and aging.

## Introduction

Recent human genome-wide association studies (GWAS) have shown that SNPs with high minor allele frequencies near the *INK4/ARF* (*CDKN2A/B*) tumor suppressor locus on chromosome 9p21 correlate with risk of atherosclerotic diseases (coronary artery disease (CAD) [1], [2], [3], [4], [5], ischemic stroke [6], [7], and abdominal aortic aneurysm[6]) and type 2 diabetes mellitus (T2DM) [8], [9], [10]. Evidence from our lab has suggested that expression of p16^INK4a^, one of the proteins encoded by the *INK4/ARF* locus, controls pancreatic islet proliferation and mass with aging [11], and therefore could plausibly explain the link to T2DM. The mechanism underlying the association of these SNPs with atherosclerotic diseases, however, is less clear.

**Figure 1:**
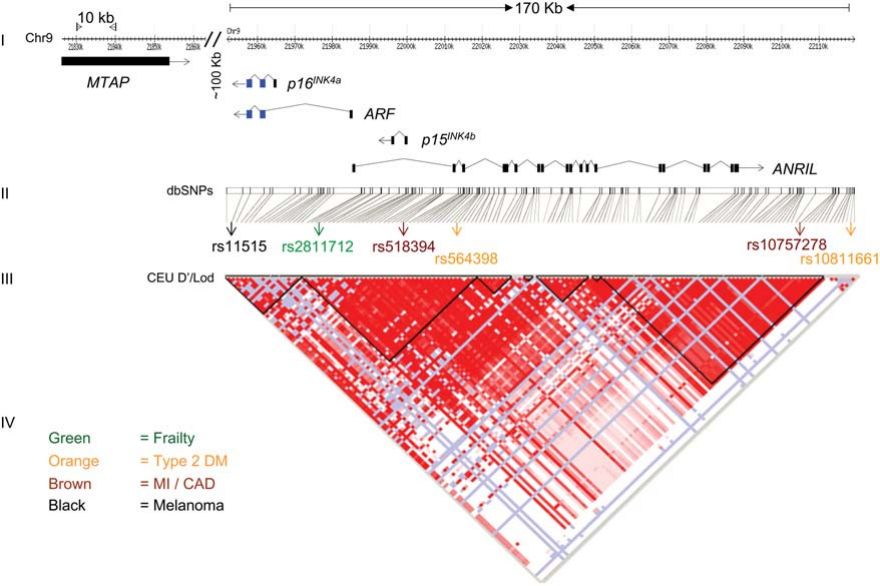
The human *INK4/ARF* locus and CAD associated SNPs. (I) *INK4/ARF*-associated transcripts and tested SNPs are shown as well as the location of *MTAP*, another gene at 9p21. Blue colored boxes indicate exons 2 and 3 shared by *p16^INK4a^* and *ARF*. The location of 9p21 SNPs (II) and linkage disequilibrium (LD) coefficient values (III) from HapMap[42] CEU (Utah residents with ancestry from northern and western Europe) are shown. The LD plot of D′/LOD was generated using Haploview software [42]. The strength of LD increases from white to blue to red: white (disequilibrium coefficient (D′) <1 and LOD score <2); blue (D′ = 1 and LOD score <2); pink (D′<1 and LOD score ≥2); and bright red (D′ = 1 and LOD score ≥2). (IV) The correlations with CAD and other age-associated conditions of the indicated SNPs are indicated by corresponding colors.

In particular, it is not known if these genetic variants are associated with altered expression of cis-encoded transcripts. The most replicated SNPs associated with strongest effect sizes for atherosclerotic disease and T2DM are >100 kb centromeric to the *INK4/ARF* locus (Fig. 1), which encodes three cell cycle inhibitors (p15^INK4b^, p16^INK4a^, ARF, reviewed in [12]) and a recently discovered putative non-coding RNA designated *ANRIL* [13]. There are no known cis-regulatory elements of the *INK4/ARF* locus located at such a distance from the locus. It has also been suggested that a more distant gene on 9p21, *MTAP* (Fig. 1), could mediate disease associations [3], [4].

In this work, we sought to determine whether there is a link between these disease-associated SNPs and expression of 9p21 transcripts. We found that individuals harboring a common SNP genotype associated with increased risk for atherosclerotic disease demonstrated markedly reduced expression of *INK4/ARF*-encoded transcripts in peripheral blood T-cells (PBTL), indicating a potentially important role for cell cycle inhibitors in atherosclerotic disease.

## Methods

### Ethics Statement

This study was approved by the University of North Carolina Institutional Review Board with all participants providing informed written consent.

### Subjects, mRNA analysis and SNP genotyping

The subject characteristics will be described elsewhere in details (YL and NES, submitted). In brief, PBTLs were obtained from 170 healthy subjects and isolated to >90% purity by Magnetic Activated Cell Sorting (MACS). MACS, RNA preparation and transcript expression analysis were performed in PBTL as described ([14], and YL and NES, submitted). Transcript expression levels are presented as log_2_-transformed expression (ΔΔC_T_, ref. [15]). Genomic DNA was prepared from buffy coats using Puregene Blood Core Kit (Qiagen) according to manufacture's instructions. All SNP genotyping was performed at the UNC Mammalian Genotyping Core using Taqman® SNP Genotyping assays by automatic genotype calling. Allele frequencies and linkage disequilibrium (LD) in studied subjects were comparable to those from HapMap (Table S1). All SNP genotypes were in Hardy-Weinberg Equilibrium (HWE) determined by Haploview software.

### Statistical analysis

Linear models with different slopes and intercepts for each genotype were used to compare the effect of genotype on *INK4/ARF* transcripts adjusting for age. ANOVA and Kruskal-Wallis tests were used to compare the mean gene expression across the genotypes (Table S2). A Student's t-test was used to compute p-values for the pair-wise comparisons between the genotypes, and p-values were adjusted using Bonferroni method for multiple comparisons (Table S2). All analysis was performed using SAS (v. 9.1.3, SAS Institute Inc).

## Results

**Figure 2:**
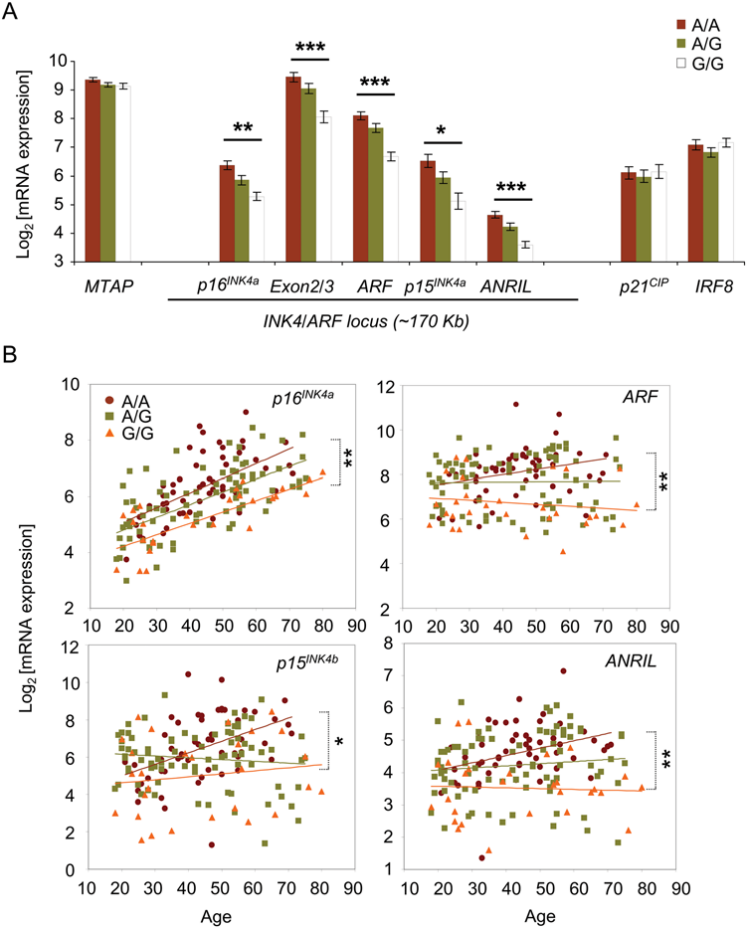
Reduced transcription of the *INK4/ARF* locus is associated with the atherosclerosis risk allele of rs10757278. (A) The risk allele (G) of rs10757278 is associated with reduced transcription of all four *INK4/ARF*-associated transcripts. Adjusted p-values (t-test) comparing mean AA vs. GG expression are shown: *p<0.001; **p<0.0005; ***p<0.0001. See also Table S2 for details of ANOVA and Kruskal-Wallis comparisons. Error bars indicate SEM. (B) Expression of genes within *INK4/ARF* locus is associated with the risk allele of rs10757278 throughout chronological age. *p<0.01 for overall slope comparison; intercepts are not compared due to significant difference in slopes. **p>0.05 for overall slope comparison and p<0.0001 for overall intercept comparison.

We selected six SNPs for genotyping based on strength of association in previous studies: two associated with atherosclerosis (rs10757278 and rs518394) [1], [2], [3], [4], two with T2DM (rs10811661 and rs564398) [8], [9], [10], one with frailty (rs2811712) [16], and one with melanoma (rs11515, in the 3′ UTR of *p16^INK4a^* and *ARF*)[17] (Fig. 1). These SNPs were also chosen to have limited LD across the locus (Fig. 1 and Table S1). While no significant associations were noted between expression of any 9p21 transcript and five of the six SNPs analyzed (not shown), a strong association was noted between *INK4/ARF* transcript expression (log_2_-transformed or ΔΔC_T_) and rs10757278 genotype (Fig. 2 and Table S2). This SNP and nearby SNPs in high LD have been reproducibly associated with atherosclerotic disease in independent studies [1], [2], [3], [4], [6], [7].

We observed significantly reduced expression of *ARF*, *p15^INK4b^*, *p16^INK4a^*, and *ANRIL* in GG vs. AA individuals (Fig. 2A and 2B). The increase in expression in AA vs. GG donors was 2.1 to 2.8-fold for all four *INK4/ARF* transcripts, with an intermediate effect seen in AG individuals for each transcript. These effects were highly significant after adjustment for multiple comparisons (p<0.0001, Table S2). While expression of *p16^INK4a^* is highly dynamic in PBTL with age, differences between genotypes were significant at all ages (Fig. 2B). Moreover, the *p16^INK4a^* and *ARF* have two shared exons (Fig. 1, reviewed in [12]), and aggregate *p16^INK4a^/ARF* expression detected with primers/probe pairs that span the shared exons (Exon2/3) were also significantly increased in AA individuals compared to GG individuals (Fig. 2A). In contrast, expression of a more distant 9p21 transcript, *MTAP*, as well as genes located on other chromosomes (*p21^CIP^* and *IRF8*) was not associated with rs10757278 genotype (Fig. 2A). As the G-allele correlates with increased risk of atherosclerotic disease [6], [18], these data indicate that decreased expression of all members of the *INK4/ARF* locus in PBTL is associated with a higher risk of atherosclerotic disease.

## Discussion

Here, we show that the expression of *INK4/ARF* locus transcripts is significantly reduced in individuals harboring a common allele of a 9p21 SNP ∼120 kb from the *INK4a/ARF* locus. This observation suggests a surprising human heterogeneity in the expression of genes that have been associated with various aspects of mammalian cancer and aging (reviewed in [19]), including a particular importance in lymphocyte aging [20]. We believe this observation can be most parsimoniously explained by the existence of a polymorphic cis-regulatory element that influences expression of the *INK4/ARF* locus and that is closely linked to rs10757278. Cis-regulatory element located more than 100 kb apart from affected open reading frames (ORFs) are well-described at other loci [21].

It is important to note that rs10757278 and linked SNPs in high LD (e.g. rs1333049) are associated with family history of atherosclerotic disease, but not other traditional risk factors including body mass index, diabetes, tobacco use, blood pressure, C-reactive protein or lipid levels [2], [22], [23]. The risk genotypes, however, are strongly associated with coronary artery calcification, premature atherosclerosis and angiographically characterized disease [2], [24], [25]. Therefore, we believe the present finding supports a direct link between *INK4/ARF* expression and atherogenesis.

We believe a reasonable argument can be made for how each of the *INK4/ARF* transcripts might modulate the earliest stages of atherosclerosis. The INK4-class cyclin dependent kinase (CDK) inhibitors, of which p15^INK4b^ and p16^INK4a^ are founding members, block cell cycle progression by inhibiting the activity of the proliferative kinases CDK4 and CDK6. A role for cell cycle inhibitors has been postulated in promoting favorable vascular remodeling and preventing pathologic intimal hyperplasia by regulating G1 to S phase progression in vascular smooth muscle cells (reviewed in [26]). In support of this notion, mice lacking p16^INK4a^ have been shown to be more sensitive to vascular intimal hyperplasia in a carotid artery injury model [27], suggesting that p16^INK4a^ protects against atherogenesis by limiting a pathologic vascular reaction to injury in some circumstances. Likewise, TGF-β signaling is thought to attenuate atherogenesis [28], 29, and the anti-proliferative effects of TGF-β signaling are in part mediated by p15^INK4b^ (ref. [30]). ARF, on the other hand, inhibits the cell cycle by activating the p53 tumor suppressor. In murine systems, Arf expression and has been shown to exert a general anti-aging effect [31], [32], presumably through an enhancement of DNA repair. Likewise, Arf expression has been shown to play a specific role in vascular biology in the development of the murine eye [33]. Together, expression of p15^INK4b^, p16^INK4a^ or ARF, either alone or in combination could plausibly retard atherogenesis, most likely by limiting pathogenic vascular proliferation.

As the putative non-coding RNA *ANRIL* spans the risk-associated SNPs, *ANRIL* might be directly affected by the unknown causative genetic variant in strong LD with rs10757278. Importantly, Polycomb group (PcG) complexes potently repress *INK4/ARF* locus expression [34], [35], [36], and cis-acting non-coding RNAs have been shown to play an important role in PcG-mediated repression at other loci (e.g. *Xist* at the inactive X-chromosome, reviewed in [37], [38]). Therefore, it is possible that altered *ANRIL* expression could potentially regulate *INK4/ARF* repression through PcG complexes, explaining why all four *INK4/ARF* transcripts are coordinately associated with rs10757278 genotype. On the other hand, *ANRIL* could also influence the expression of other genes in trans- as has been recently reported for *HOTAIR* at *Hox* loci [39].

Importantly, however, it is likely the effects of *INK4/ARF* expression on the development of atherosclerosis do not result from their expression in PBTL, but rather, observations in PBTL may serve as a surrogate for *INK4/ARF* expression in other tissues. As cis-regulatory elements may act as enhancers in one tissue and repressors in another, it is also possible that genetic variants in LD with rs10757278 genotype might be associated with increased *INK4/ARF* transcript expression in other tissues of greater relevance to atherogenesis (e.g. endothelial progenitors or vascular smooth muscle cells). Several investigators have argued that cellular senescence, which is induced by p16^INK4a^, promotes atherosclerosis (e.g. [40], [41]), and we do not believe the present results are necessarily inconsistent with that view. Carefully designed murine studies will serve to elucidate the effects of *INK4/ARF* expression on atherogenesis.

In summary, our data suggests that a common genetic variant in strong LD with rs10757278 at chromosome 9p21 influences the expression of the *INK4/ARF*-associated transcripts, but not *MTAP*, in PBTL. These observations provocatively suggest a crucial role for cell cycle inhibition in the development of atherosclerotic disease in humans.

## Supporting Information

Table S1 (2.37 MB TIF)

Table S2 (2.42 MB TIF)
