# Supplementary figures and images for: INK4/ARF Transcript Expression Is Associated with Chromosome 9p21 Variants Linked to Atherosclerosis
Source: PLoS One. 2009 Apr 3;4(4):e5027. doi: 10.1371/journal.pone.0005027 (PMC2660422; doi:10.1371/journal.pone.0005027)

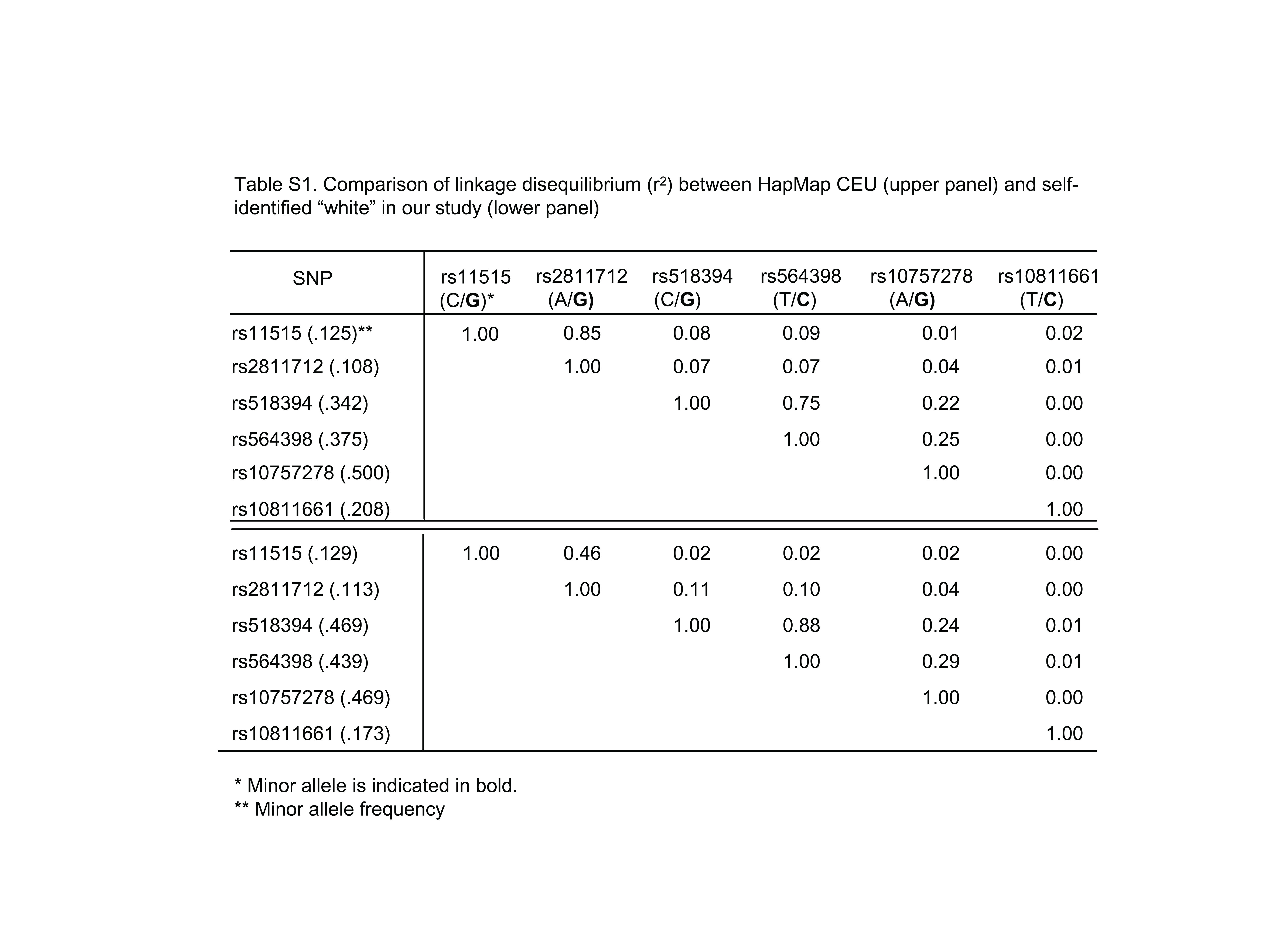

Supplement: Table S1 — (2.37 MB TIF) [file pone.0005027.s001.tif]
